# The Milky Way

**Published:** 1881-09

**Authors:** 


### THE MILKY WAY.

Light travels at the rate of 186,000 miles per second. Yet it is calculated that the light from the nearest star to us in the Milky Way occupies about three years in reaching the earth, and that the light of the most distant will not arrive at the earth in less than 1,500 years.

The Milky Way is a wide, irregular ribbon of stellar clouds which crosses the sky in all its width. It is, indeed, nothing more, according to astronomers, than the greatest length of this immense lens of stars to which we belong. If the whole sky does not appear nebulous in every direction, it is because the nebula to which we belong is not spherical, but of a lenticular form, and that in the thickness of the lens there is less depth and fewer stars than in the direction of the diameter. From the spot on which we are placed, if our sight pass through the greatest length, it meets stars on stars indefinitely, because there is an immense expanse from the point where we are to the edges of the flattened nebula. But if our sight turn aside from the equatorial plane toward the sides, it meets with fewer stars as it gets farther distant.

All the stars which sparkle in the sky during a dark night belong to a single cluster, to a single nebula, the Milky Way marking its longitudinal direction. The stars are not isolated in an absolute manner, at random, in the deserts of space; they form part of a whole; the sun which lights us is one of them, and they are counted by millions in a gigantic group, analogous to the distant clusters. Instead of seeing a diffused glimmer, an indistinct light in the Milky Way, the telescope separates the stars which compose it and shows that it is formed of an innumerable multitude of stars very irregularly connected.

The idea which we must form of the Milky Way is then very different from that which appearances presents to us, and from that with which the ancients contented themselves. From the beginning of ages, from the first observations of an elementary astronomy, the semi-luminous train which crosses the sky was noticed, and the ruling mythology adorned it with images.

William Herschel, with the powerful telescope made with his own hands, resolved, toward the end of the last century, to count the stars comprised in this zone ; he addressed himself to his task and divided his work into portions. His long perseverance was crowned with success. By a careful comparison of the parts where the condensation of stars attains its maximum with those where it attains its minimum, and by an examination of the extent occupied by these immense rings, the great observer found that the Milky Way did not inclose less than 18,000,000 of stars !

This is not the total number of which it is composed, as this does not refer to the lateral portions of this gigantic mass, and all the stars of the heavens situated on one side and on the other of the plane of the greatest condensation are not included in this enumeration. What is the real extent occupied by this collection of suns ? The number of stars which compose it, and the relative distances from each other, comprises for this extent a number which the mind cannot well receive without being prepared for it, a number which it cannot appreciate without making great efforts to grapple it. We will not give the distance in leagues, because an immense continuation of leagues exceeds the limits of even the vision of the mind ; it is better to take the measure used constantly for astronomical units. Now, the extent of the Milky Way, at its greatest length would be measured by a ray of light which, traveling 186,000 miles per second, would travel in a straight line, and without stopping, for 15,000 years.

Thus, as we are ourselves near the centre of this nebula, when in the field of a powerful telescope we observe the little distant stars situated in the depths of the Milky Way, our retina receives the impression of a luminous ray, which started 7,000 or 8,000 years ago from a sun analogous to ours, and forming part of the same group.

If such be the extent of the nebula of which we are an infinitessimal constituent part, are not the other nebulæ scattered in space also as rich and vast. The Milky Way is not unique; many of the nebulæ of the universe are so many milky ways, more or less similar to our own. Some may be less vast ; others may be vaster still, seeing that in the domain of the infinite, spaces goes for nothing. It is best for us, then, to take the middle course, and to think that the pale and diffused nebulæ which seem to tremble in the distance in unfathomable immensities, are milky ways peopled with as many suns as our own.

